# Characterization and functional expression of the natriuretic peptide system in human lens epithelial cells

**Published:** 2010-04-09

**Authors:** Patrick R. Cammarata, Brittany Braun, Slobodan D. Dimitrijevich, Jessica Pack

**Affiliations:** 1 Department of Cell Biology and Anatomy, University of North Texas Health Science Center at Fort Worth, Fort Worth, TX; 2 Department of Integrative Physiology, University of North Texas Health Science Center at Fort Worth, Fort Worth, TX

## Abstract

**Purpose:**

The family of natriuretic peptides (NPs); atrial natriuretic peptide (ANP), brain natriuretic peptide (BNP), and C-type natriuretic peptide (CNP) as well as three associated receptors (NPRs); natriuretic peptide receptor A (NPR-A), natriuretic peptide receptor B (NPR-B), and natriuretic peptide receptor C (NPR-C) has never been documented in human lens epithelial cells. The study described herein was designed to demonstrate both expression and functionality of components of the natriuretic peptides and natriuretic peptide receptors in the human lens epithelial cell line, HLE-B3 and in normal human lens epithelial cell cultures (nHLE).

**Methods:**

Reverse transcriptase-polymerase chain reaction (RT-PCR) along with confirmation by DNA sequencing and real-time quantitative RT–PCR was used to identify and demonstrate expression of mRNA for the natriuretic peptide family. Authentication of protein expression of the natriuretic peptide receptors was determined by using formaldehyde-fixed, Saponin-permeabilized cells (HLE-B3) or methanol:acetone-fixed and permeabilized cells (nHLE) using conventional immunofluorescence techniques. Enzyme-linked immunosorbent assay was used to determine cyclic GMP (cGMP) activity as stimulated by exogenous addition of natriuretic peptides.

**Results:**

Using RT–PCR with confirmation by DNA sequencing and real-time quantitative RT–PCR, HLE-B3 cells were shown to express mRNA for *ANP*, *BNP*, and *CNP* along with their associated receptors. Conventional immunofluorescence on the permeabilized cells confirmed positive diffuse staining indicating the presence of the three natriuretic peptide receptors in both HLE-B3 and nHLE cells. All three natriuretic peptides educe a cGMP response in the rank order CNP>>ANP≈BNP indicating that the natriuretic peptide family is functional in HLE-B3 cells.

**Conclusions:**

The data indicates that *ANP*, *BNP*, and *CNP* and natriuretic peptide receptor transcripts are expressed and are functional in human lens epithelial cells. The cellular expression of NPs and NPRs, as well as the demonstration that all three NPs activate guanylyl cyclase suggests a potential role in maintaining lens epithelial cell homeostasis.

## Introduction

The natriuretic peptide (NP) family has evolved for the homeostatic purposes of volume, osmosis, and pressure regulation of the circulatory system. The natriuretic peptide system comprises three structurally related peptides: atrial natriuretic peptide (ANP), brain natriuretic peptide (BNP) and C-type natriuretic peptide (CNP). Corresponding with the peptides are three individual natriuretic peptide receptors: natriuretic peptide receptor A (NPR-A), natriuretic peptide receptor B (NPR-B), and natriuretic peptide receptor C (NPR-C) [1].

As stated in a recent review by Potter et al. [2], “ANP and BNP are circulating peptides that activate the transmembrane guanylyl cyclase, natriuretic peptide receptor-A. CNP activates a related cyclase, natriuretic peptide receptor-B. Both receptors catalyze the synthesis of cGMP, which mediates most known effects of natriuretic peptides. A third natriuretic peptide receptor, natriuretic peptide receptor-C, clears natriuretic peptides from the circulation through receptor-mediated internalization and degradation.” The receptor may participate in mediating some of the cellular actions of the natriuretic peptides via coupling to Gi proteins and negative modulation of adenylyl cyclase activity [3]. The predominant action of NPR-C, at least in the cardiovascular system, seems to be the modulation of circulating and local natriuretic peptide concentrations that are available to bind NPR-A and NPR-B [4]. Natriuretic peptides play an important role in the regulation of cardiovascular homeostasis maintaining blood pressure and extracellular fluid volume. The classical endocrine effects of natriuretic peptides to modulate fluid and electrolyte balance and vascular smooth muscle tone are complemented by autocrine and paracrine actions that include (among other actions), cytoprotective anti-ischemic effects [5].

Molecular evidence has been reported for the presence and functionality of the natriuretic peptide system in several ocular systems, including: ciliary epithelium [6], trabecular meshwork [7], and the human retina [8,9] and more recently, in normal and transformed corneal epithelial cells (Slobodan D. Dimitrijevich, personal communication). In the eye, natriuretic peptides have been shown to regulate intraocular pressure and stimulate guanylate cyclase. The three natriuretic peptide receptors, but particularly the NPR-C receptor, have been reported to adjust the NP concentration of the aqueous humor [10]. It is equally possible that the natriuretic peptide system may be involved in the regulation of lens volume within the lens (for a current review of the regulation of lens volume, refer to [11]). Lens transparency is dependent on an ordered tissue architecture which must be well maintained. Any disruption within the architecture of the lens can cause light to scatter resulting in difficulty in imaging and eventually lens cataract. Hence the volume of the lens epithelial cells and fiber cells that make up the bulk of the lens needs to be tightly regulated if lens transparency is to be preserved. Lens volume is regulated via regulation of ion concentrations. Decreases in volume can be controlled by the loss of K^+^ and Cl^-^ ions and obligatory water loss. Channels and transporters involved in expulsion of these ions include of K^+^ and Cl^-^ channels as well as potassium chloride cotransporters (KCCs). In contrast, increases in lens volume can be driven by the intracellular accumulation of K^+^, Na^+^, and Cl^-^ ions. The natriuretic peptides and receptors may be involved, in an as yet unknown manner, with this volume regulation.

More recently, a unique mechanism for the cellular action of the natriuretic peptides has been described by Garcia-Dorado et al. [12]. They have reported that, “Reperfusion injury may cause myocardial cell death and limit the benefit achieved by restoration of coronary artery patency in patients with acute myocardial infarction. The mechanism includes altered Ca(2+) handling with cytosolic and mitochondrial Ca(2+) overload, Ca(2+)- and ATP-dependent hypercontraction, cytoskeletal fragility, mitochondrial permeability transition and gap junction-mediated propagation of cell death, as well as alterations in non-cardiomyocyte cells, in particular platelets and endothelial cells. cGMP favorably modulates all of these mechanisms but cGMP synthesis is altered in reperfused cardiomyocytes and endothelial cells by mechanisms that are only partially understood. Stimulation of cGMP synthesis during initial reperfusion by means of natriuretic peptides has been found protective in different animal models and in patients. Moreover, increasing evidence indicates that cGMP is an important step in signal transduction of endogenous cardioprotection. Thus, the cGMP pathway appears as a key element in the pathophysiology of myocardial ischaemia-reperfusion and as a promising therapeutic target in patients with acute myocardial infarction.”

With particular respect to cGMP synthesis and its relationship to mitochondrial permeability transition, the Cammarata laboratory is geared toward the elucidation of the signaling pathways involved in lens mitochondrial protection (i.e., mitoprotection). We have previously shown that in the human lens epithelial cell line, HLE-B3, extracellular signal-regulated kinase (ERK), and more specifically phospho-extracellular signal-regulated kinase 2 (pERK2), plays a crucial role in mitoprotection [13], as it does in ischemic myocardium [14]. But the mechanisms of lens mitoprotection are far more complex than just activation of ERK and cGMP is likely to play a role via the phosphatidylinositol-3 kinase/Akt (PI3-K/Akt) pathway.

The lens exists in a natural state of hypoxia [15]. Oxygen concentration in the cortical region of the lens is below 5% and may be as low as 1% in the nuclear zone [15-17]. This state of severe oxygen deprivation, an environment to which the lens has adapted, would be detrimental to most tissues, raising several important biologic questions as to how the lens is able to cope with hypoxia. It is clearly evident that the lens has developed unique survival mechanisms enabling it to thrive in a chronically hypoxic environment and to oppose oxidative injury, all the while maintaining normal mitochondrial function and resisting apoptosis. However, the specific adaptive mechanisms enabling the lens to survive hypoxia have not been delineated. Our hypothesis regarding the signaling mechanisms involved in lens mitoprotection generally incorporate two signal transduction pathways. One branch involves ERK activation [13] and glycogen synthase kinase-3β (GSK-3β) inhibition, relieving the interference of mitoprotection by an extramitochondrial GSK isoform [18]. The second branch involves sequential activation of PI3-kinase and Akt which initiates the remainder of the cytosolic pathway: 1. endothelial nitric oxide synthase (eNOS) phosphorylation activates nitric oxide (NO) production; 2. NO stimulates guanylyl cyclase (in the lens epithelium it is likely that natriuretic peptides coupled to their receptors do this as well); 3. the cGMP produced by guanylyl cyclase activates protein kinase G (PKG), which causes mitochondrial ATP-sensitive K(^+^) channel (mitoK_ATP_) opening. For different perspectives on these signaling pathways the reader is referred to several excellent articles [19-21].

ERK activation is functional in the lens epithelial cell and linked to mitoprotection [13]. The influence on mitoprotection by subsequent downstream effects after Akt activation may also play a role but this remains to be determined. To that end, eNOS activity is a crucial component of the latter scheme as previously described by Miki et al. [14]. In the course of our ongoing studies, we became aware that eNOS was greatly diminished or missing from the lens epithelial cell. In light of the fact that eNOS is a crucial player in the PI3-kinase/Akt mitoprotective scheme via the generation of cGMP [14], and given the fact that natriuretic peptides provide mitoprotection via the generation of cGMP [12], the study presented herein was designed as a first demonstration of the presence and functionality of the natriuretic peptides and their associated natriuretic peptide receptors in a human lens epithelial cell line, HLE-B3.

## Methods

### Cell culture

An SV-40 virus immortalized human lens epithelial cell line, HLE-B3, was used for the majority of experiments [22]. The cells were maintained in Eagle’s minimal essential medium (MEM) containing 20% fetal bovine serum (FBS) and 2 mM L-glutamine, nonessential amino acids, and 0.02 g/l gentamycin solution and maintained at 37 °C and 5% CO_2_ as previously described by Flynn, et al. [13,23]. The immortalized human lens epithelial cell line, SRA 01/04 [24] was generously provided by Frank Giblin (Oakland University, Eye Research Institute, Rochester, MI) and Marc Kantorow (Florida Atlantic University, Biomedical Sciences Department,  Boca Raton, Fl).

For the majority of experiments, HLE-B3 cells were grown to confluence in 20% FBS MEM and stepped down into 2% FBS MEM for a period of 24 h. This was followed by a step down into serum-free MEM for an additional 24 h before collection of cell samples. With one experiment, the cells were placed in a hypoxia chamber (5.0% O_2_) in serum-free MEM for up to 3 h. Following the hypoxic incubation, the medium was replaced with fresh oxygenated serum-free MEM and the cells returned to normoxia (ambient oxygen) for reoxygenation collections.

Whole globes (male, 2 months old; donor tissue from eye banks) were incubated in serum-free Dulbecco's minimal essential medium (DMEM) containing 10,000 units of penicillin and 10 mg streptomycin/ml (Sigma-Aldrich, St. Louis, MO) at 4 °C for 30 min followed by additional 30 min incubation in DMEM with half the concentration of penicillin and streptomycin. The globes were rinsed in sterile PBS, pH 7.4. The lenses were carefully excised from the ciliary processes, and a slit was cut across the lens capsule at the equator and the capsule peeled off, discarding the cortex/nucleus. Remaining interior lens fragments were removed from the capsule by careful irrigation with serum-free DMEM. Thereafter, the cells were cultured in tissue culture flasks and subsequently plated onto collagen-coated coverslips or tissue culture plastic in 20% FBS MEM for experimentation.

### Reverse transcriptase-polymerase chain reaction

Reverse transcriptase-polymerase chain reaction (RT–PCR) was performed on populations of HLE-B3 cells. The cells were lysed, collected and purified according to instructions supplied with the Illustra RNAspin Mini kit (GE Healthcare, Piscataway, NJ). RNA concentration was determined spectophometrically and RT–PCR was performed to obtain cDNA. RT–PCR was performed with cDNA and synthesized primers (Sigma-Aldrich Inc., St. Louis, MO) using previously published primers for the natriuretic peptides and their receptors as reported by Rollín et al. [8]. The PCR products were electrophoresed on 2% agarose gels, the bands excised, and sent out for sequencing (Northwoods DNA, Inc., Solway, MN).

Quantitative RT–PCR was performed on synthesized cDNA in a Smart Cycler thermocycler using a Brilliant Sybr Green core reagent kit (Stratagene, La Jolla, CA) and the natriuretic peptide and natriuretic peptide receptor primer pairs were used in the RT–PCR experiments at a final concentration of 200 µM. Relative increases in mRNA are based upon average ΔC_T_ values calculated from comparisons to an actin standard and to baseline C_T_ values determined from cells maintained under normal (20% FBS MEM, 5.0% CO_2_) conditions.

### Western blot analysis

Western blotting was performed as described by Flynn, et al. [13]. Briefly, total cell lysates for HLE-B3 cells were collected following subjection to different conditions by rinsing with PBS, pH 7.0, and subsequent cell lysis with a hot extraction lysis buffer, pH 6.8, consisting of 0.12M Tris-HCl, 4% SDS, and 20% glycerol. This was conducted by direct addition of 250 µl of lysis buffer heated to 100 °C to the cell monolayers. Cell lysates were immediately scraped and collected in 1.5 ml Eppendorf tubes and followed by sonication and snap freezing in liquid nitrogen. Samples were stored at −80 °C until use. Protein concentration was determined using the DC protein assay (Bio-Rad, Hercules, CA). SDS (Laemmli) buffer (3×) was added to the lysates, which were subsequently boiled for 10 min. Proteins were resolved by electrophoresis on 12% SDS-polyacrylamide gels (10 µg protein/lane). Proteins were then transferred onto nitrocellulose using a TE 22 Mini Transfer Tank (Hoefer Inc. Holliston, MA).

For western blot analysis, nitrocellulose membranes were blocked with 0.1% BSA in Tris-buffered saline (TTBS) for 30 min. The membranes were then washed four times with 0.1% BSA and 0.02% Tween-20 in TTBS at 15 min per wash. The membranes were probed overnight at 4 °C with primary antibody for eNOS (Assay Designs, Ann Arbor, MI) or actin (Santa Cruz Biotechnology, Santa Cruz, CA). The following day, blots were washed 4× for 15 min each in TTBS and then incubated with goat anti-rabbit secondary antibodies (Santa Cruz Biotechnology, Santa Cruz, CA) for 1 h at room temperature. Blots were again rinsed 4× for 15min in TTBS and proteins were detected using a SuperSignal west pico chemiluminescent kit from Pierce (Rockford, IL). Probed membranes were imaged using an alphafluor imager (Alpha Innotech Corporation, San Leandro, CA). eNOS electrophoresis standard (Cayman Chemical Company, Ann Arbor, MI) was included to authenticate the western blot data.

### Immunocytochemistry

Immunocytochemistry was conducted as previously described by Flynn et al. [23]. In brief, HLE-B3 cells were seeded onto coverslips in 35 mm dishes and maintained in MEM with 20% FBS for 24 h at 37 °C, 5% CO_2_. Cells were subsequently stepped down to MEM with 2% FBS for 24 h followed by 24 h in serum-free MEM. Prior to the step down into serum-free MEM cells were rinsed with sterile 0.05 M PBS, pH 7.0.

To prepare for imaging, the serum-free MEM was removed, the cells were rinsed with sterile PBS and fixed in 1% paraformaldehyde in PBS at 4 °C for 30 min. The fixative was subsequently removed and the cells rinsed twice (10 min each rinse) in 0.05 M PBS, pH 7.0, containing 50 mM NH_4_Cl. Washing was followed by incubation with blocking buffer which contained 0.05% Saponin and 2% BSA in PBS for 20 min at room temperature. Cells were incubated with primary antibody for NPR-A, NPR-B, and NPR-C at a 1:1,000 dilution (Santa Cruz Biotechnology) at 4 °C overnight. The coverslips were then washed with washing buffer 4× at 5 min per wash and incubated with a fluorescent secondary antibody, Alexa Fluor 488 Goat-anti-rabbit (Invitrogen Corporation, Carlsbad, CA), at a 1:500 dilution. Controls for immunohistochemical staining included a pure rabbit IgG control and control containing no primary antibody. Imaging was conducted on a Zeiss LSM 410 (Carl Zeiss MicroImaging, Inc., Thornwood, NY) at 40× magnification.

For the normal human lens epithelial cells, approximately 15,000 cells (passage 2) were plated on glass coverslips (12 cm^2^; Thermofisher; Fisher Scientific, Pittsburgh, PA) and cultured in DMEM containing 20% FBS. After 3 days of culture, the coverslips were rinsed in PBS, and fixed in methanol:acetone −1:1 (v/v) for 30 min at 4 °C. The cells were then rinsed with PBS and buffered saline (PBS, 0.256 g/l NaH_2_PO_4_ H_2_O, 1.19 g/l Na_2_HPO_4_, 8.76 g/l NaCl, pH 7.4; for 30 min), followed by distilled water washes (3×). Thereafter, the cells were stained (overnight at 4 °C) with 4’,6-diamino-2-phenylindole (200 nm, DAPI, 10 min) and mounted on glass slides, and rinsed with distilled water (3×), followed by incubation at 4 °C, overnight, with primary antibody diluted in PBS as above. After rinsing in PBS, containing Tween-20 (0.1%, 3× for 10 min each), cells were incubated with secondary antibody at room temperature (RT, 1.5 h) as above and rinsed in PBS +Tween-20 (0.1%, 3× for 10 min each). The specimens were finally rinsed in PBS (3× for 10 min each) followed by distilled water (30 min) and imaging was conducted on a Zeiss LSM 410” at 40× magnification. Control for immunocytochemical staining included no primary antibody.

### cGMP assay

For the cGMP assay, cells were plated into 35 mm dishes and grown overnight to near confluence in 20% FBS MEM. On day two, the cells were rinsed twice with serum-free media and then incubated in serum-free MEM containing 25 µM 3-isobutyl-1-methylxanthine (IBMX) for 15 min at 37 °C. Following the incubation period ANP, BNP, or CNP (Sigma-Aldrich, Saint Louis, MO) in concentrations of 0.1 µM, 1.0 µM, or 5.0 µM were added to the individual plates and cells were further incubated for 10 min. Cells were lysed and the activity assay was performed according to manufacturer’s instructions (Cayman Chemical Company, Ann Arbor, MI). Spectrophometric readings were completed using a Spectra Max Pro (model 190) with Molecular Probes software (Molecular Devices, Sunnyvale, CA). Statistical analysis was completed with the use of the cGMP assay’s manufacturer supplied excel spreadsheet.

## Results

### Expression of mRNA for the natriuretic peptides and their receptors in HLE-B3 cells

**Figure 1:**
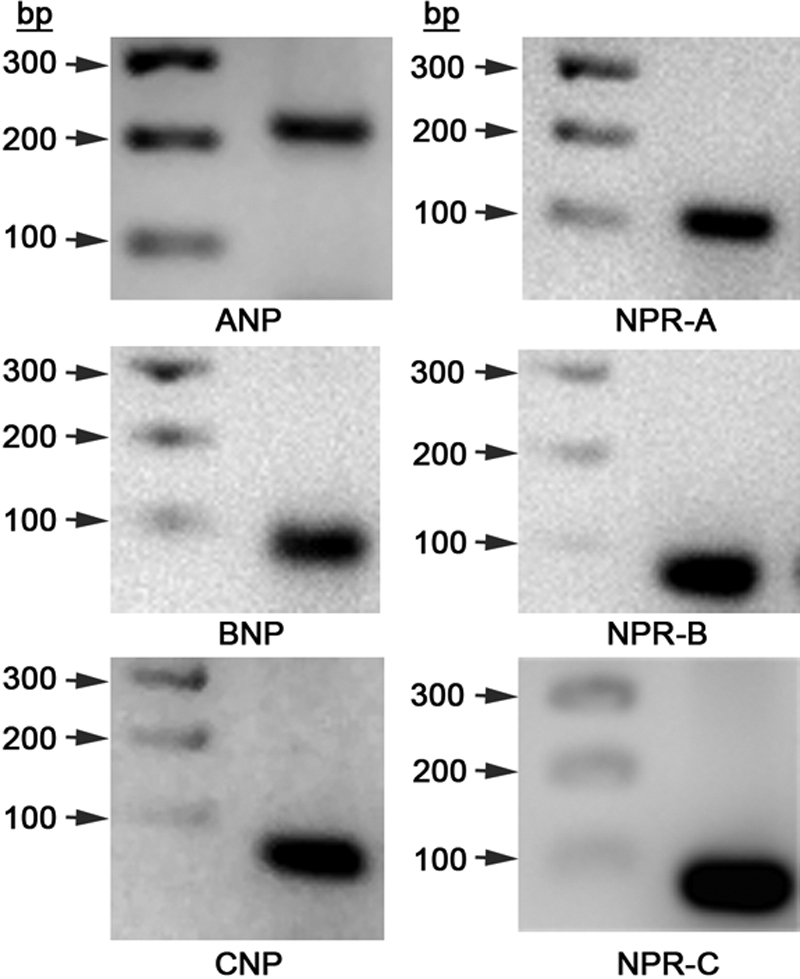
Reverse Transcriptase-PCR analysis of the natriuretic peptides (*ANP*, *BNP*, and *CNP*) and receptors (*NPR-A*, *NPR-B*, and *NPR-C*) in HLE-B3 cells. Total RNA was isolated from HLE-B3 cells and subjected to RT–PCR for cDNA synthesis. Polymerase chain reactions were run with the synthesized cDNA with the primer pairs for each of the three natriuretic peptides and natriuretic peptide receptors. PCR products were run on 2.5% agarose gel alongside a 100 bp step ladder (Promega Corporation, Madison, WI). All products resolved in the expected location.

RT–PCR was used to determine *ANP*, *BNP*, and *CNP* as well was *NPR-A*, *NPR-B*, and *NPR-C* mRNA expression in HLE-B3 cells (Table 1). All PCR products resolved at the expected fragment size (Figure 1). The bands were excised from the agarose gels and submitted for sequencing to further verify the accuracy of the experiment and confirm positive identification by sequence of the PCR products. The resulting sequences were entered into NCBI Basic Local Alignment Search Tool (BLAST) which confirmed with 100% homology the three natriuretic peptides and associated natriuretic peptide receptors.

**Table 1:** Natriuretic peptide and receptor oligonucleotide primer pairs.

**Gene (GeneBank accession)**	**Primer sequence (Forward/Reverse)**	**Location**	**PCR product (bp)**
Actin, beta (*ACTB*; NM_001101)	5′-CATCCTCACCCTGAAGTACC-3′ 5′-GTACAGGGATAGCACAGCCT-3′	273–513	241
Natriuretic peptide precursor A (*NPPA*; NM_006172)	5′-GATTTCAAGAATTTGCTGGACCAT-3′ 5′-TTGCTTTTTAGGAGGGCAGATC-3′	209–435	227
Natriuretic peptide receptor A (*NPR-A*; NM_000906.3)	5′-GCAAAGGCCGAGTTATCTACATC-3′ 5′-AACGTAGTCCTCCCCACACAA-3′	1131–1228	98
Natriuretic peptide precursor B (*NPPB*; NM_002521)	5′-CGGGTTACAGGAGCAGCG-3′ 5′-CTCCAGGGATGTCTGCTCCA-3′	225–297	73
Natriuretic peptide receptor B (*NPR-B*; NM_003995)	5′-CGGGAGGATGGACTTCGA-3′ 5′-CATGACAACCAGCCCAGTTACA-3′	1072–1146	75
Natriuretic peptide precursor C (*NPPC*; NM_024409)	5′-AGCGTGGGCTCGCCTT-3′ 5′-CTTGTTGGCTCCTTTGTATTTGC-3′	249–309	61
Natriuretic peptide receptor C (*NPR-C*; NM_000908)	5′-GGAAGACATCGTGCGCAATA-3′ 5′-GATGCTCCGGATGGTGTCA-3′	950–1028	79

Primers used for reverse transcriptase and quantitative real time PCR were previously published by Rollín et al. [8].

Our results with real-time quantitative RT–PCR verified the expression of the natriuretic peptide system mRNA (*ANP*, *BNP*, *CNP*, *NPR-A*, *NPR-B*, and *NPR-C*) in human lens epithelial cells. Serum deprivation resulted in an approximate threefold increase in expression of *ANP* and *CNP* with respect to cells maintained in 20% FBS, while *BNP* expression decreased (Table 2). *NPR-A* and *NPR-B* expression increased an approximate 2 and fourfold under serum starvation conditions, while *NPR-C* expression was essentially unchanged.

**Table 2:** Quantitative RT–PCR analysis of mRNA expression for the natriuretic peptides and receptors in HLE-B3 cells.

**Experiment**	**ANP**	**BNP**	**CNP**	**NPR-A**	**NPR-B**	**NPR-C**
Serum free	3.30±0.13	0.42±0.16	2.65±0.17	2.18±0.34	4.15±0.08	1.30±0.22

mRNA expression levels were measured by quantitative RT–PCR in an experiment in which HLE-B3 cells were subjected to serum deprivation. Independent triplicate populations of cells were collected. Analysis of quantitative RT–PCR data was based upon calculating ΔC_T_ values using a β-actin control and a baseline standard of mRNA expression at 20% FBS MEM. Values represent an n=6 and are expressed relative to the respective baselines (20% FBS MEM) which are assigned a value of 1.0. Values represent the mean±standard error.

### Immunocytochemical imaging for identification of natriuretic peptide receptor synthesis in HLE-B3 cells and normal human lens epithelial cells

**Figure 2:**
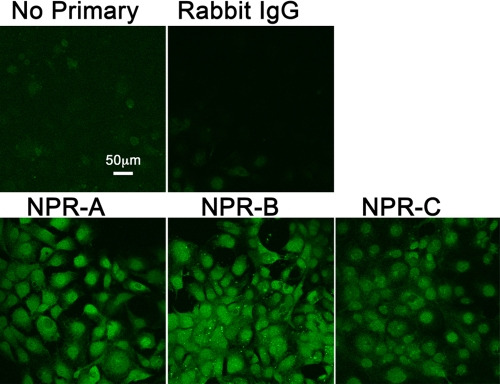
Immunoflourescent analysis of natriuretic peptide receptor expression in HLE-B3 cells. Immunoflourescence and confocal microscopy was used to determine expression of natriuretic peptide receptors on HLE-B3 cells. Control images included HLE-B3 cells incubated with no primary antibody but with a goat-anti-rabbit secondary and cells incubated with rabbit IgG as the primary antibody. The data are typical of images representative of eight random fields of view per receptor taken from two independent populations of cells. The scale bar equals 50 microns.

**Figure 3:**
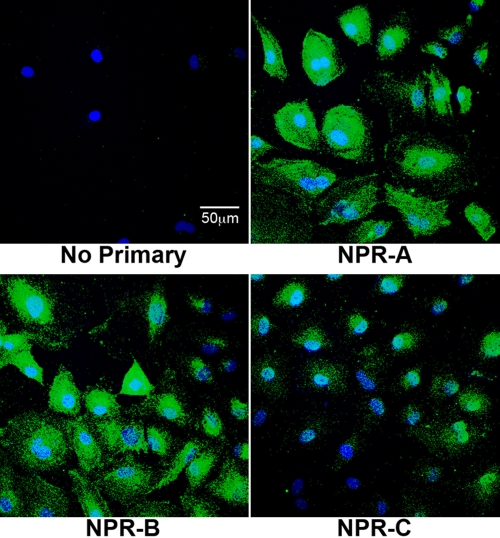
Immunoflourescent analysis of natriuretic peptide receptor expression in normal human lens epithelial cells (nHLE). Immunoflourescence and confocal microscopy was used to determine expression of natriuretic peptide receptors in cultured normal human lens epithelial cells. Control image included nHLE cells incubated with no primary antibody but with a goat-anti-rabbit secondary. The data are typical of images representative of three or four random fields of view per receptor taken from a population of cells derived from whole globes (passage 2). The scale bar equals 50 microns.

Immunocytochemistry and confocal microscopy were used to identify and verify the presence of the natriuretic peptide receptors in HLE-B3 cells. HLE-B3 cells stepped down into serum-free MEM displayed positive diffuse immunostaining for each of the three receptors: NPR-A, NPR-B, and NPR-C (Figure 2). Immunocytochemistry and confocal microscopy was repeated using a second fixation method on normal human lens epithelial cell cultures (nHLE). Immunostaining of the nHLE cells also displayed a positive diffuse pattern for each of the three receptors: NPR-A, NPR-B, and NPR-C (Figure 3). As with the virally-transformed cells, immunostaining was more intense for NPR-A and NPR-B as was the density of immunostaining for NPR-C with the cultured nHLE cells. Although this is a likely depiction of the relative distribution of NPR-A, NPR-B, and NPR-C, it should be pointed out that the use of antibody immunostaining is not, of itself, a reliable quantitative measurement. While the data does not permit assignment of the receptors to specific localization of a particular organelle or whether the immunostaining reflects localization to the inner (and for that matter, outer) plasma membrane surface, it does confirm the intracellular presence (and so, by inference, synthesis) of the natriuretic peptide receptors in both virally-transformed human lens epithelial cells and normal human lens epithelial cells.

### cGMP activity assay

**Figure 4:**
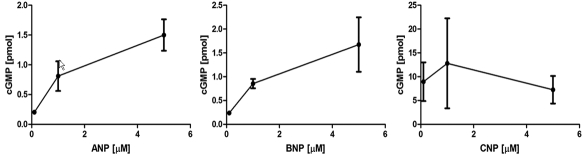
Analysis of natriuretic peptide induced cyclic GMP production in HLE-B3 cells. Cyclic GMP activity was measured to determine functionality for the natriuretic peptide system in HLE-B3 cells. Values shown are representative of means of two independent populations of cells examined in triplicate samples (n=6). Each point on the graph represents the mean±standard error.

To determine functionality of the natriuretic peptide system, we tested whether the addition of ANP, BNP, and CNP to the cells stimulated guanylate cyclase activity. HLE-B3 cells were subjected to bolus addition of natriuretic peptides in varying concentrations: 0.1 µM, 1.0 µM, and 5.0 µM and incubated for 10 min. Addition of bolus natriuretic peptides in varying concentrations elicited a cGMP response in HLE-B3 cells for all three peptides at all concentrations (Figure 4).

Although all three peptides elicited a cGMP response, the response provoked by addition of CNP was significantly higher than that of ANP and BNP. Under minimum concentration of peptide (0.1 µM) CNP displayed a 30 to 40 fold higher cGMP production when compared to ANP and BNP. Whereas ANP and BNP appeared to have a dose dependent effect on cGMP production, CNP addition elicited maximal cGMP response at all three concentrations of peptide tested. It is therefore likely that a considerably lower concentration of CNP would elicit a cGMP response, attesting to its enhanced effectiveness to educe a cGMP response. The order of potency of the peptides in production of a cyclic GMP response is CNP»BNP≈ANP. It did not escape our attention that this finding precisely mirrors the published results of Ortego and Coca-Prados [6] for natriuretic peptide-stimulated cGMP response in the ciliary process and that of Pang et al. [7] in the ciliary muscle and trabecular meshwork. In both prior studies, CNP elicited a strikingly higher cGMP response than either ANP or BNP.

### Protein expression for eNOS

**Figure 5:**
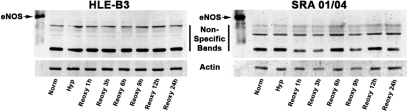
eNOS production in human lens epithelial cell lines HLE-B3 and SRA 01/04. Protein expression for eNOS was measured by western blot analysis with HLE-B3 and SRA 01/04 cells subjected to hypoxia followed by reintroduction of oxygen. Western blots were run with an actin control and the lanes were equally loaded at a concentration of 10 µg protein per lane. Note that authentic eNOS standard was detected with the commercial antibody.

Protein expression for eNOS was evaluated using western blotting. Expression profiles were examined with HLE-B3 and SRA 01/04 cells maintained in serum-free normoxia, hypoxia, and upon reintroduction of ambient oxygen through a duration of up to 24 h. β-actin was used as a control. eNOS expression was undetectable for the collected cell samples in either the HLE-B3 cell line or the SRA cell line and the antibody employed was authenticated by positive affirmation with eNOS standard (Figure 5).

## Discussion

There are a scant few studies demonstrating the functional existence of the natriuretic peptide system in regions of the human eye [6-9]. This is the first report of the presence and functionality of the natriuretic peptide system in human lens epithelial cells.

The present study is the first report by real-time quantitative RT–PCR of the expression of the NP system mRNA for all three natriuretic peptides and receptors in cultured human lens epithelial cells. Although most NPs and NPRs had expression levels consistently higher under serum starvation conditions relative to control, *BNP* decreased approximately 50% and *NPR-C* remained relatively unchanged when subjected to this stress condition (Table 2). The factors that can affect mRNA expression of the natriuretic peptides and receptors in ocular systems are largely uncharacterized.

Western blot analysis to detect changes in the expression profile of the natriuretic peptides and the natriuretic peptide receptors was inconclusive and the natriuretic peptides were undetectable by immunocytochemical imaging (data not shown). There are several potential reasons for the inability to detect natriuretic peptides by western blot and immunohistochemical imaging. Chiefly, is the likelihood that they behave in a manner similar to neurotransmitters, in that they are rapidly synthesized and degraded, resulting in low steady-state concentrations. For example, Ortego and Coca-Prados [6] had to resort to the use of radioimmunoassay to achieve a level of sensitivity capable of detecting natriuretic peptides in their systems. While we were able to demonstrate that mRNA for the natriuretic peptides and natriuretic peptide receptors is transcribed in the lens epithelial cell (Table 2), we do not yet know the fate of these transcripts after their production. Indeed, while it is conceivable that the natriuretic peptides might function in an autocrine or paracrine manner, they might be rapidly synthesized and degraded or rapidly flushed from the aqueous humor. For that matter, synthesis of the encoded proteins may not generally occur in the lens epithelium but instead the natriuretic peptides might be produced by other ocular cells (ciliary epithelium, retina) and then exert their effects on the surface natriuretic peptide receptors of the lens epithelium.

Nevertheless, we were able to demonstrate the functionality of the NP system in HLE-B3 cells via the generation of cGMP upon addition of exogenous natriuretic peptides across a broad concentration range (Figure 4). CNP evoked the greatest increase in cGMP production in the lens cells. A possible reason for CNP producing such a large induction of cGMP production may very well be that the NPR-B receptor subtype is the primary receptor in these ocular systems [10], and based upon real-time quantitative mRNA expression, may also be in the lens epithelium. We suggest that there is an as yet unappreciated role for CNP, demonstrated now in several ocular systems. It is worthwhile to restate that in non-ocular systems, it appears that ANP and BNP play the predominant role via their natriuretic and diuretic effects whereas, as documented now with several ocular systems, it is evident that CNP is more efficient in cGMP generation over that of ANP and BNP.

We suggest an alternative role for the natriuretic peptide system in lens epithelium other than volume regulation, that of mitochondrial protective agents. Stressful conditions, which can include serum starvation and hypoxia, increase formation of reactive oxygen species (ROS). Intracellular accumulation of ROS and calcium can provoke a change in mitochondrial permeability transition (MPT) through a high-conductance pore in the inner part of the mitochondrial membrane. MPT initiates intracellular cascades culminating in cell death [25] and MPT leads to a loss of ATP synthesis [26]. The mitochondria of the lens epithelial cell are endowed with protective mechanisms to prevent cell death from occurring [13,23]. Protective signals are transmitted to the mitochondria via signaling pathways including those using cGMP, which may interfere with mechanisms of cell death. Specific details of this chemistry have been modeled in an excellent review by Garcia-Dorado et al. [12]. With respect to the NPs potential to serve as mitoprotectants in the lens epithelium, all three peptides elicited an increase in cGMP production with CNP showing the highest potency (Figure 4). We suggest that the function of the natriuretic peptide system in the lens epithelium is to compensate for the apparent lack of (or greatly diminished amount of) eNOS in the lens epithelium, as demonstrated by its failure to be detected by western blot analysis in two lens epithelial cell lines (Figure 5). NO production leads to nitric oxide-sensitive guanylyl cyclase activation producing cGMP ultimately leading to MPT pore stabilization and thus mitochondrial protection [12,14]. A plausible role of the natriuretic peptide system in the lens epithelial cell may well be to act as a secondary pathway to compensate for the lack of sufficient or adequate levels of NO production (as generated via eNOS) as a means to generate cGMP and offer protection to the mitochondria against depolarization, although this remains to be definitively established in the lens epithelial cell model. However, until proven otherwise, it must be stated that it is equally likely that given the apparent lack of lenticular eNOS and the low expression of the lenticular natriuretic peptides, despite the fact that exogenous addition of NPs educed a cGMP response, that neither system plays a biologically functional role with respect to lens epithelial cell mitoprotection. Experiments are currently in progress to determine the influence of the PI3-kinase/Akt-cGMP- mitoK_ATP_ channel pathway on mitochondrial protection and whether this pathway is functionally prevalent in lens epithelial cells.

## References

[1] Koller KJ Goeddel DV Molecular Biology of the Natriuretic Peptides and their Receptors. Circulation 1992 86 1081 8 1327579 10.1161/01.cir.86.4.1081

[2] Potter LR Yoder AR Flora DR Antos LK Dickey DM Natriuretic peptiods: their structures, receptors, physiologic functions and therapeutic applications. Handb Exp Pharmacol 2009 191 341 66 19089336 10.1007/978-3-540-68964-5_15 PMC4855512

[3] Murthy KS Teng BQ Zhou H Jin JG Grider JR Makhlouf GMG (i-1)/G(i-2)-dependent signaling by single-transmembrane natriuretic peptide clearance receptor. Am J Physiol Gastrointest Liver Physiol 2000 278 G974 80 10859228 10.1152/ajpgi.2000.278.6.G974

[4] Matsukawa N Grzesik WJ Takahashi N Pandey KN Pang S Yamauchi M Smithies O The natriuretic peptide clearance receptor locally modulates the physiological effects of the natriuretic peptide system. Proc Natl Acad Sci USA 1999 96 7403 8 10377427 10.1073/pnas.96.13.7403 PMC22098

[5] Woodard GE Rosado JA Natriuretic peptides in vascular physiology and pathology. Int Rev Cell Mol Bio 2008 268 59 93 18703404 10.1016/S1937-6448(08)00803-4

[6] Ortego J Coca-Prados M Functional expression of components of the natriuretic peptide system in human ocular nonpigmented ciliary epithelial cells. Biochem Biophys Res Commun 1999 258 21 8 10222228 10.1006/bbrc.1999.0573

[7] Pang IH Shade DL Matsumoto S Steely HT DeSantis L Presence of functional type B natriuretic peptide receptor in human ocular cells. Invest Ophthalmol Vis Sci 1996 37 1724 31 8759339

[8] Rollin R Mediero A Roldan-Pallares M Fernandez-Cruz A Fernandez-Durango R Natriuretic peptide system in the human retina. Mol Vis 2004 10 15 22 14737067

[9] Cao LH Yu YC Zhao JW Yang XL Expression of natriuretic peptides in rat muller cells. Neurosci Lett 2004 365 176 9 15246543 10.1016/j.neulet.2004.04.090

[10] Fernandez-Durango R Moya FJ Ripodas A de Juan JA Fernandez-Cruz A Bernal R Type B and type C natriuretic peptide receptors modulate intraocular pressure in the rabbit eye. Eur J Pharmacol 1999 364 107 13 9932712 10.1016/s0014-2999(98)00828-0

[11] Donaldson PJ Chee KS Lim JC Webb KF Regulation of lens volume: Implications for lens transparency. Exp Eye Res 2009 88 144 50 19091312 10.1016/j.exer.2008.05.011

[12] Garcia-Dorado D Agullo L Sartorio CL Ruiz-Meana M Myocardial protection against reperfusion injury: The cGMP pathway. Thromb Haemost 2009 101 635 42 19350105

[13] Flynn JM Lannigan DA Clark DE Garner MH Cammarata PR RNA suppression of ERK2 leads to collapse of mitochondrial membrane potential with acute oxidative stress in human lens epithelial cells. Am J Physiol Endocrinol Metab 2008 294 E589 99 18171912 10.1152/ajpendo.00705.2007

[14] Miki T Miura T Tanno M Nishihara M Naitoh K Sato T Takahashi A Shimamoto K Impairment of cardioprotective PI3K-Akt signaling by post-infarct ventricular remodeling is compensated by an ERK-mediated pathway. Basic Res Cardiol 2007 102 163 70 16944359 10.1007/s00395-006-0622-3

[15] McNulty R Wang H Mathias RT Ortwerth BJ Truscott RJ Bassnett S Regulation of tissue oxygen levels in the mammalian lens. J Physiol 2004 559 883 98 15272034 10.1113/jphysiol.2004.068619 PMC1665185

[16] Holekamp NM Shui YB Beebe DC Vitrectomy surgery increases oxygen exposure to the lens: a possible mechanism for nuclear cataract formation. Am J Ophthalmol 2005 139 302 10 15733992 10.1016/j.ajo.2004.09.046

[17] Shui YB Beebe DC Age-dependent control of lens growth by hypoxia. Invest Ophthalmol Vis Sci 2008 49 1023 9 18326726 10.1167/iovs.07-1164 PMC2585417

[18] Murphy E Steenbergen C Does inhibition of glycogen synthase kinase protect in mice. Circ Res 2008 103 226 8 18669927 10.1161/CIRCRESAHA.108.181602 PMC2617729

[19] Budas GR Mochly-Rosen D Mitochondrial protein kinase Cε (PKCε): emerging role in cardiac protection from ischaemic damage. Biochem Soc Trans 2007 35 1052 4 17956277 10.1042/BST0351052

[20] Di Lisa F Canton M Menabo R Kaludercic N Bernardi P Mitochondria and cardioprotection. Heart Fail Rev 2007 12 249 60 17516167 10.1007/s10741-007-9028-z

[21] Downey JM Davis AM Cohen MV Signaling pathways in ischemic preconditioning. Heart Fail Rev 2007 12 181 8 17516169 10.1007/s10741-007-9025-2

[22] Andley UP Rhim JS Chylack LT Jr Fleming TP Propagation and immortalization of human lens epithelial cells in culture. Invest Ophthalmol Vis Sci 1994 35 3094 102 8206728

[23] Flynn JM Dimitrijevich SD Younes M Skliris G Murphy LC Cammarata PR Role of wild-type estrogen receptor-beta in mitochondrial cytoprotection of cultured normal male and female human lens epithelial cells. Am J Physiol Endocrinol Metab 2008 295 E637 47 18577698 10.1152/ajpendo.90407.2008

[24] Ibaraki N Chen SC Lin LR Okamoto H Pipas JM Reddy VN Human lens epithelial cell line. Exp Eye Res 1998 67 577 85 9878220 10.1006/exer.1998.0551

[25] Garlid KD Costa AD Quinlan CL Pierre SV Dos Santos P Cardioprotective signaling to mitochondria. J Mol Cell Cardiol 2009 46 858 66 19118560 10.1016/j.yjmcc.2008.11.019 PMC2683183

[26] Halestrap AP Brennerb C The adenine nucleotide translocase: A central component of the mitochondrial permeability transition pore and key player in cell death. Curr Med Chem 2003 10 1507 25 12871123 10.2174/0929867033457278

